# Use of molecular markers in identification and characterization of resistance to rice blast in India

**DOI:** 10.1371/journal.pone.0176236

**Published:** 2017-04-26

**Authors:** Manoj Kumar Yadav, Aravindan S., Umakanta Ngangkham, H. N. Shubudhi, Manas Kumar Bag, Totan Adak, Sushmita Munda, Sanghamitra Samantaray, Mayabini Jena

**Affiliations:** ICAR-National Rice Research Institute, Cuttack, Odisha, India; Fujian Agriculture and Forestry University, CHINA

## Abstract

Rice blast disease caused by *Magnaporthe oryzae* is one of the most destructive disease causing huge losses to rice yield in different parts of the world. Therefore, an attempt has been made to find out the resistance by screening and studying the genetic diversity of eighty released rice varieties by National Rice Research Institute, Cuttack (NRVs) using molecular markers linked to twelve major blast resistance (*R*) genes viz *Pib*, *Piz*, *Piz-t*, *Pik*, *Pik-p*, *Pikm Pik-h*, *Pita/Pita*-2, *Pi2*, *Pi9*, *Pi1* and *Pi5*. Out of which, nineteen varieties (23.75%) showed resistance, twenty one were moderately resistant (26.25%) while remaining forty varieties (50%) showed susceptible in uniform blast nursery. Rice varieties possessing blast resistance genes varied from four to twelve and the frequencies of the resistance genes ranged from 0 to 100%. The cluster analysis grouped the eighty NRVs into two major clusters at 63% level of genetic similarity coefficient. The PIC value for seventeen markers varied from 0 to 0.37 at an average of 0.20. Out of seventeen markers, only five markers, 195R-1, Pi9-i, Pita3, YL155/YL87 and 40N23r corresponded to three broad spectrum *R* genes viz. *Pi9*, *Pita/Pita2* and *Pi5* were found to be significantly associated with the blast disease with explaining phenotypic variance from 3.5% to 7.7%. The population structure analysis and PCoA divided the entire 80 NRVs into two sub-groups. The outcome of this study would help to formulate strategies for improving rice blast resistance through genetic studies, plant-pathogen interaction, identification of novel *R* genes, development of new resistant varieties through marker-assisted breeding for improving rice blast resistance in India and worldwide.

## Introduction

Rice (*Oryza sativa* L.) is the staple food for more than half of the world’s population and provide more than 19% of the calories consumed by the world population [1]. It is predicted that the world population will exceed 8 billion people by 2025 and to meet these global food demands, the production of grain needs to increase up to 50% more by the year 2025 [2]. However, a rigorous yield loss that has affected the rice cultivation is accounted to biotic and abiotic stresses [3]. Besides, the emergence of new races with the changing climate are the major issues that address the requirement for sustainable crop development and resistance to biotic stresses. In the recent times, with the advances made in the area of molecular markers, the tracking of the genes for resistance is possible by following the path of markers that are linked/tagged to each gene for resistance, thus making simple for the identification of plants containing more genes.

Rice blast is the most important fungal rice disease, which is caused by the fungus *Magnaporthe oryzae*. This disease has been reported to occur in more than 85 countries. The yield losses caused by rice blast were nearly 30–50% under conducive environmental conditions [4,5]. It can infect rice plant right from seedlings to adult plant stages affecting leaves, nodes, collar, panicles and roots. Although, chemical control of the disease is viable, it remains economically unfeasible for resource poor farmers and is environmentally hazardous at high application rates. The development and deployment of rice varieties introgressed with major resistance genes is the most cost-effective and environment-friendly approach to combat the menace of blast disease. Resistance to *M*. *oryzae* is known to follow gene for gene theory, where major resistance (*R*) gene is effective in preventing infection by a race of *M*. *oryzae* containing the corresponding avirulence (*Avr*) gene [6]. So far, over 100 blast resistance genes have been identified, of them, 45% are from *japonica* cultivars, 51% are from *indica* cultivars and the remaining 4% are from wild species of rice [7]. These *R* genes are distributed all over the 12 rice chromosomes except chromosome 3 [8,9]. Out of them, 25 have been cloned and characterized [10,11]. The majority of the blast *R* genes are dominant and qualitative, except the recessive gene *pi21*, and a few (*pi21* and *Pb1*) were reported as quantitative in nature [12]. The rapid change in the virulence characteristics of *M*. *oryzae* populations pose a continuous threat to the effectiveness of existing blast resistant varieties [13]. Therefore, strategies for developing durable resistance mediated by major resistance gene have been proposed and widely adopted [14]. These strategies depend upon cautious classification of the resistance spectrum of the appropriate *R* genes against the pathogen races and combining them in such a way that the ‘gene pyramiding’ is effective against wide spectrum of blast races [15,16,17]. Pyramiding of resistance genes using conventional breeding procedure is time consuming and less efficient because blast races carrying individual avirulence genes for the identification of the corresponding resistance gene are usually not present in nature [18].

The use of different resistant rice varieties in the cultivation of rice, could be the most active, and cost effective way to reduce the adverse effect of the blast disease. Thus, the improvement of resistant varieties against blast disease is one of the most important objectives in the rice breeding plan [19,20]. Although, a considerable number of high yielding rice varieties have been released in India by NRRI for diverse ecologies along with available good agronomic practices, these are not fully exploited due to the lack of information/knowledge on resistance to biotic stresses. Therefore, the present study aimed at assessing 80 varieties including 2 rice hybrids developed by NRRI, Cuttack, Odisha, India to find out the resistance against leaf blast and simultaneously characterized them for eleven major blast resistance (*R*) genes using available tightly linked/gene based markers conferred for broad spectrum resistance to many races of blast pathogen. This is the first attempt to characterize the NRRI released rice varieties at molecular level which will help in utilization of these popular rice varieties in blast endemic areas for different ecologies.

## Material and methods

### Plant material

A set of 80 NRVs were selected and collected from the National Gene Bank, NRRI, Cuttack (S1 Table). This set consisted of different varieties released from 1970 to 2014 for eight different ecologies being cultivated throughout India and many rice growing regions of the world. Two additional genotypes, CO39 and HR-12 were included as blast susceptible checks.

### Disease reaction in uniform blast nursery

NRVs were evaluated for their spectrum of reaction against the leaf blast at the Uniform Blast Nursery (UBN) at the research farm of NRRI, Cuttack (85°55′48″ E longitudes and 20°26′35″ N latitude). The evaluation was conducted in two replications during dry and wet seasons, 2015–16. A 50-cm-long row of each entry was planted in nursery beds with a row spacing of 10 cm. One row each of susceptible check (CO39 and HR-12) was planted after every five entries and also along the borders to facilitate the uniform spread of the disease. Observations on the blast reaction of the lines were recorded after 25 days of sowing and continued at 5 days interval until 40^th^ day of sowing or when the susceptible checks had disease symptom (85%), whichever occurred earlier. Spectrum of disease reaction was scored visually on a 0–9 scale following the Standard Evaluation System (SES), IRRI, Philipines (2002). The lines with scores of 0–3 were considered highly resistant, 4–5 as moderately resistant, and 6–9 as susceptible. Whenever there was different score values between the replications, only the higher score was considered for evaluation.

### Genomic DNA isolation

Genomic DNA was isolated from young leaf tissue following the cetyltrimethyl ammonium bromide (CTAB) method [21] with minor modifications. The quantity and quality of purified genomic DNA was estimated on 0.8% agarose gels electrophoresis and NanoDrop ND-1000 Spectrophotometer (Thermofisher scientific, USA). The DNA samples were later diluted with nuclease-free water to the concentration of 20 ng/μl for PCR amplification.

### Molecular screening for rice blast *R* genes

For molecular screening, the NRVs were genotyped for the presence of 12 major blast resistance genes viz. *Pib*, *Piz*, *Piz*-*t*, *Pik*, *Pik-p*, *Pikm*, *Pik-h*, *Pita/Pita*-2, *Pi2*, *Pi9*, *Pi1*, a-2 and *Pi5*. A total of 17 markers available for above twelve genes were collected and used for molecular screening. The details of information of the primer pairs for seventeen markers used in the present study are listed in Table 1. The physical location of the seventeen markers were depicted along the corresponding chromosomes (S1 Fig). PCR amplification was carried out in a 20 μl reaction volume containing 20 ng template DNA, 0.2 μM of each of dNTP, 0.2 μM of each forward and reverse primers, 1.5 mM MgCl_2_, 1X Taq buffer (10 mM Tris-HCl, 50 mM KCl, pH 8.3) and 1U of *Taq* DNA polymerase (DreamTaq, Thermo Scientific, USA). The PCR programme was set up as follow: initial denaturation of 5 min at 94°C; followed by 35 cycles of denaturation for 45 sec at 94°C, primers annealing for 45 sec at varied temperatures (Table 1), and extension for 2 min at 72°C, followed by a final extension for 10 min at 72°C. For scoring the marker loci, the amplified PCR products were separated by electrophoresis along with a 100 bp DNA ladder (BR Biochem Life Sciences, India) in 2–3% agarose gels stained with ethidium bromide. After electrophoresis, the gels were documented under UV using gel documentation system (AlphaImager, USA). The PCR amplified fragments were scored as presence (1) or absence (0). All PCR reactions for each primer were repeated twice to cross-check the scoring data.

**Table 1:** List of marker used for blast resistance genes.

Sl.No.	*R* Gene	Chr no.	Marker used	Positive control	Primer sequence		Type of marker	Annealing temp.	Expected size (bp)	References
					Forward (5`-3`)	Reverse (5`-3`)				
1.	*Pib*	2	Pb28	IRBLB-b	GACTCGGTCGACCAATTCGCC	ATCAGGCCAGGCCAGATTTG	SNP	60	388	[30]
2.	*Piz*	6	Z56592	IRBLZ-Fu	GGACCCGCGTTTTCCACGTGTAA	AGGAATCTATTGCTAAGCATGAC	SNP	60	292	[30]
3.	*Piz-t*	6	Zt56591	IRBLZT-t	TTGCTGAGCCATTGTTAAACA	ATCTCTTCATATATATGAAGGCCAC	SNP	60	257	[30]
4.	*Pik*	11	K39512	IRBLZK-Ka	GCCACATCAATGGCTACAACGTT	CCAGAATTTACAGGCTCTGG	SNP	60	112	[30]
5.	*Pik-p*	11	K3957	IRBLZKP-k60	ATAGTTGAATGTATGGAATGGAAT	CTGCGCCAAGCAATAAAGTC	SNP	60	148	[30]
6.	*Pik-h*	11	Candidate gene marker	IRBLKH-k3	CATGAGTTCCATTTACTATTCCTC	ACATTGGTAGTAGTGCAATGTCA	Gene based marker	55	1500	[38]
7.	*Pi9*	6	195R-1	IRBL9-w	ATGGTCCTTTATCTTTATTG	TTGCTCCATCTCCTCTGTT	STS	56	2,000	[45]
			9-Pro		TGATTATGTTTTTTATGTGGGG	ATTAGTGAGATCCATTGTTCC	Allele specific	50	111 for Pi2/Piz-t allele, 128 for Pi9 allele	[46]
			Pi9-i		GCTGTGCTCCAAATGAGGAT	GCGATCTCACATCCTTTGCT	FNP	54		[47]
8.	*Pi2*	6	Pi-2GM	IRBLZ5-CA	GATTTAGTTCAGGAAAACACTC	TGGAAGCCTCATTGATCATC	Gene specific	55	2344	[31]
			2-LRR		CGTTGTATAGGACAGTTTCATT	AATCTAGGCACTCAAGTGTTC	Allele specific	50	436/439	[46]
			Pi2-i		CAGCGATGGTATGAGCACAA	CGTTCCTATACTGCCACATCG	FNP	52	450/282	[47]
9.	*Pita/Pita-2*	12	Pita3	IRBLTA-KI	AGTCGTGCGATGCGAGGACAGAAAC	GCATTCTCCAACCCTTTTGCATGCAT	SNP	59	861	[30]
			YL155/YL87	-	AGCAGGTTATAAGCTAGGCC	CTACCAACAAGTTCATCAAA	Dominant	55	1042	[48,49]
10.	*Pi1*	11	RM1233	C101LAC	GTGTAAATCATGGGCACGTG	AGATTGGCTCCTGAAGAAGG	SSR	55	170/155	[18]
11.	*Pi5*	9	40N23r	IRBLz5-M	TGTGAGGCAACAATGCCTATTGCG	CTATGAGTTCACTATGTGGAGGCT	InDel	55	700/480	[50]
12.	*Pikm*	11	k2167	IRBLKMTS	CGTGCTGTCGCCTGAATCTG	CACGAACAAGAGTGTGTCGG	InDel	55	619/300	[51]

### Allele scoring and diversity analysis

The binary matrix representing different alleles of the seventeen markers which were scored as binary data whether present (1) or absent (0) was used for estimation of genetic distance and similarity coefficients. Genetic similarities were estimated from the matrix of binary data using Jaccard’s coefficient. The resultant similarity matrix was subjected to construct dendrogram using Sequential Agglomerative Hierarchical Nesting (SAHN) based Unweighted Pair Group Method with Arithmetic Means (UPGMA) to their genetic relationships [22] using NTSYS-PC version 2.0 [23]. Polymorphism information content (PIC) value of the marker was estimated using the program POWERMARKER Ver3.25 [24].

### Statistical analysis for blast resistance genes

To understand the significant genetic association of blast resistant genes with the blast disease, the genetic associations was studied using the general linear model (GLM) function in TASSEL5 software [25]. The GLM of Tassel 5 software was conducted with permutations of 1000. Analysis of the population structure was carried out using the program STRUCTURE version 2.3.4 [26]. The number of subgroups (K) in the population was determined by running the programme at different K values from K = 1 to K = 10, with 5 independent iterations per K using the admixture model and correlated allele frequencies with 200,000 burn-in period and 200,000 MCMC. Then, the peak value of ΔK was used to determine the optimal K according to the method described by Evanno et al. [27] using STRUCTURE HARVESTER programme [28]. The PCoA (Principal Coordinate Analysis) was computed using the binary data of markers through GenAlEx 6.502 [29]. The values of the eigen vectors obtained were plotted in a scatter graph taking the first and the second principal components as the axes. The *R* genes genotypic data was used to conduct analysis of molecular variance (AMOVA) to partition the total molecular variance and significant F_ST_ using GenAlEx 6.502 [29].

## Results

### Phenotyping of leaf blast disease

**Fig 1:**
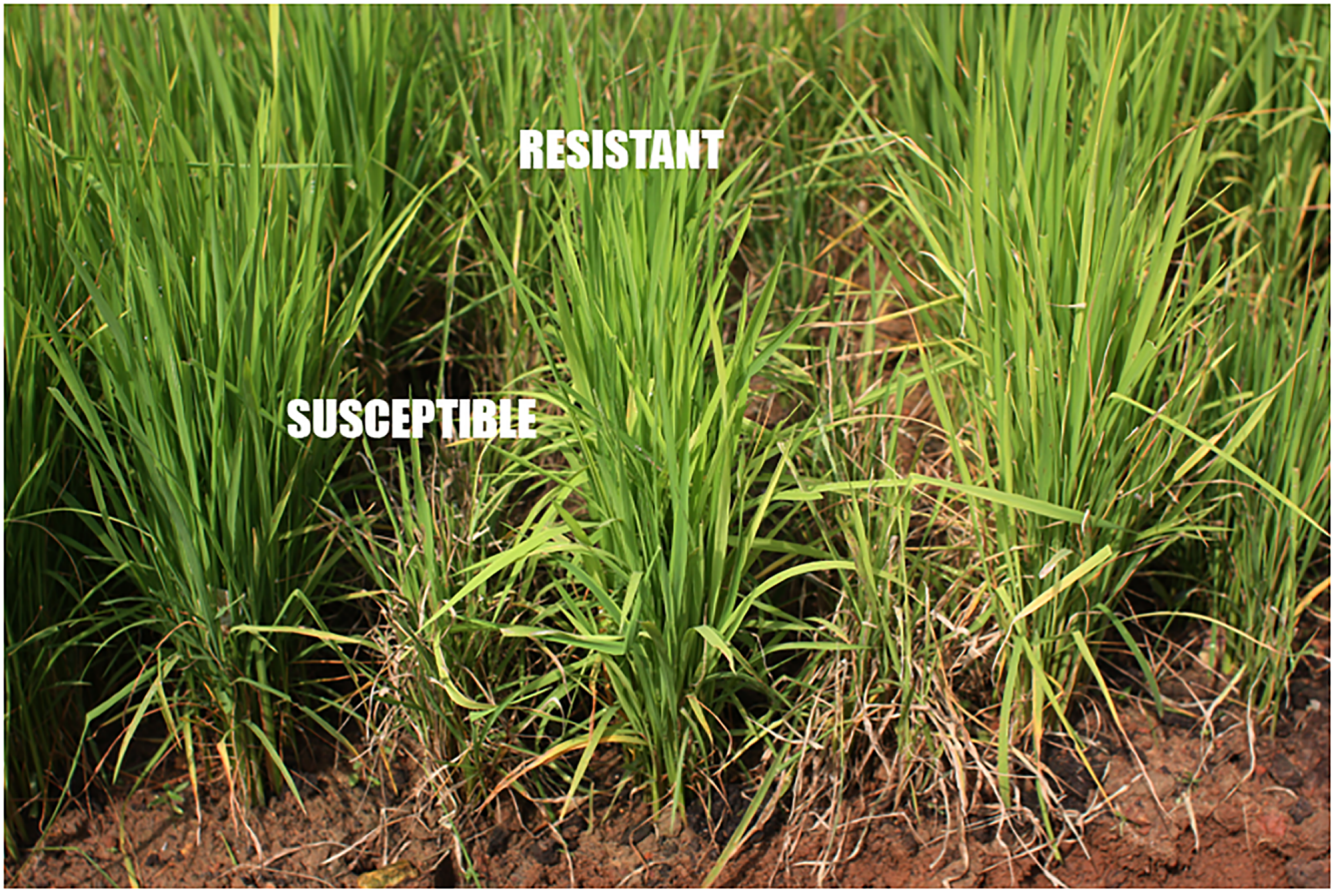
Reaction of rice genotypes to leaf blast in uniform blast nursery (UBN) at Cuttack.

Based on the screening scores against leaf blast in UBN, all the 80 NRVs were classified in such a way that nineteen varieties (23.75%) were found highly resistant (Score 0–3), twenty-one varieties (26.25%) were moderately resistant (score 4–5), while forty varieties (50%) were found susceptible (score 6–9) (Fig 1, Table 2). Based on ecology, all the 80 NRVs were classified into eight different ecologies such as irrigated (30), shallow lowland (15), upland (10), medium deep water logged (9), coastal saline (6), aerobic (5), deep water (3) and boro ecotype (2). In irrigated ecology, there were fifteen NRVs each which were found to be resistant and susceptible, respectively. Similarly, in the case of shallow low land, eight were found to be resistant and seven were susceptible. In upland, out of 10 varieties, four were resistant and six were susceptible. Unlike above ecologies, there was only one resistant variety compared to eight susceptible varieties in medium deep water logged ecology. In the coastal ecology, out of six varieties, four were found to be resistant and two were susceptible. However, in the case of stress or unfavourable ecology such as aerobic, deep water and boro, there was no susceptible varieties as compared to the above different ecologies (Table 2).

**Table 2:** Evaluation of NRRI released varieties (NRVs) for leaf blast resistance in the uniform blast nursery.

S. No.	Ecology	No. of NRVs	Resistant (0–3 score)	Moderately Resistant (4–5 score)	Susceptible (6–9 score)
**1.**	Irrigated	**30**	Satya Krishna, Chandrama, Abhishek, Sarasa and CR Dhan 300 **(5)**	Improved Lalat, Radhi, HUE, Geetanjali, Naveen, Shaktiman, Kalinga-I, CR Dhan 305 Rajalaxmi and Ajay **(10)**	CR Dhan 303, Supriya, CR Dhan 907, Phalguni, Improved Tapaswini, Saket-4, Maudamani, Kalinga-II, Tapaswini, Ratna, CR Dhan 304, CR Dhan306, Khitish, Indira and Udaya **(15)**
**2.**	Shallow low land	**15**	Sumit, Savitri, Reeta and Samalei **(4)**	NuaDhusara, NuaKalajeera, CR Dhan 701 and Ketekijoha **(4)**	NuaChinikamini, Padmini, PoornaBhog, Dharitri, Moti, Pooja and Swarna sub-1 **(7)**
**3.**	Upland	**10**	Satyabhama, and Sahbhagidhan **(2)**	Hazaridhan **(1)**	Virender, Annada, Anjali, Kamesh, Sattari, Heera and Kalyani-II **(7)**
**4.**	Medium deep water logged	**9**	Panidhan **(1)**	NIL	Durga, Utkalprabha, CR 1014, Hanseswari, CR Dhan 501, Sarala, Gayatri and Varshadhan **(8)**
**5.**	Coastal Saline	**6**	Lunishree **(1)**	LunaBarial, Luna Sampad and Luna Suvarna **(3)**	LunaSankhi and Sonamani **(2)**
**6.**	Aerobic	**5**	CR Dhan 205, CR Dhan 202 and CR Dhan 204 **(3)**	Pyari **(1)**	CR Dhan 201 **(1)**
**7.**	Deep water	**3**	Jalamani, and Jayanti Dhan **(2)**	CR Dhan 500 **(1)**	NIL
	Boro	**2**	Chandan **(1)**	CR Dhan 601 **(1)**	NIL
	**Total**	**80**	**19**	**21**	**40**

### Genetic diversity of blast resistant *R* genes

**Fig 2:**
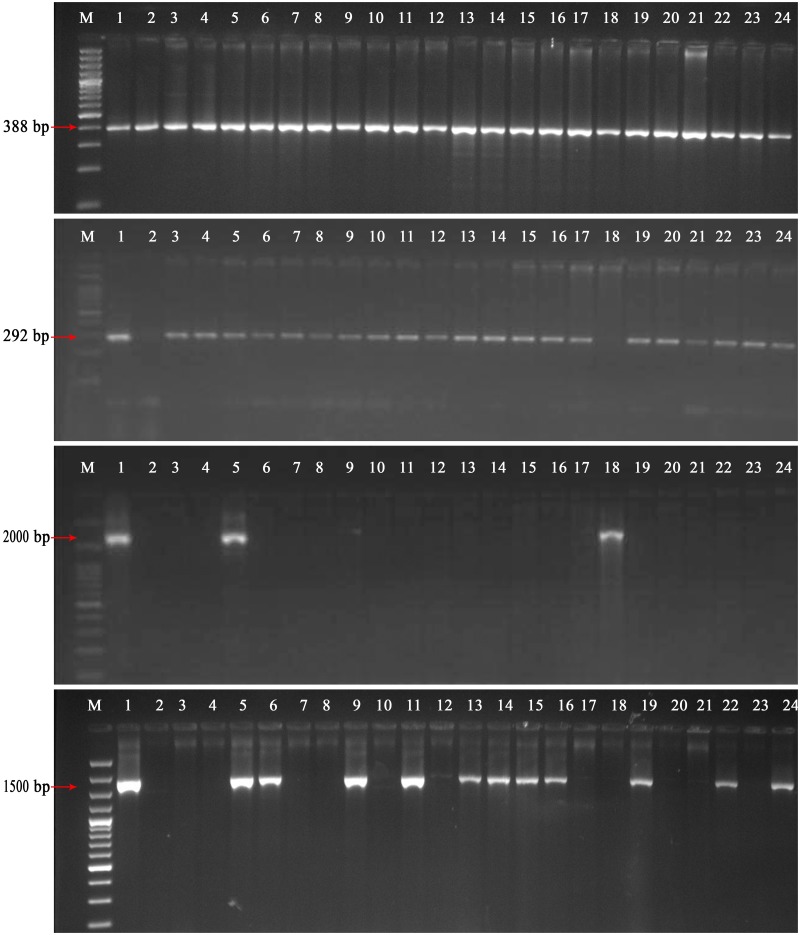
**Agarose gel photograph of 22 NRVs, to know the presence or absence of** A) 388 bp of *Pib* rice blast resistance gene amplified with SNP marker Pb28; B) 292 bp of *Piz* rice blast resistance gene amplified with SNP marker z56592; C) 2000 bp of *Pi9* rice blast resistance gene amplified with gene based STS marker; D) 1500 bp of *Pik-h* rice blast resistance gene amplified with gene based marker. M denotes ladder; lane 1 positive control; lane 2 CO39 (negative control); lanes 3–24: rice varieties.

**Fig 3:**
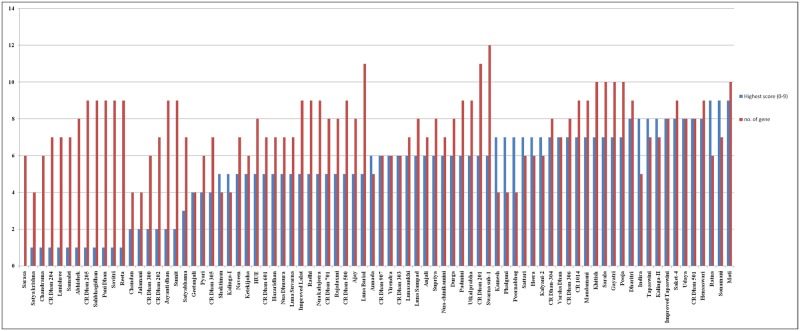
Reaction of rice genotypes to leaf blast in uniform blast nursery (UBN) at Cuttack and number of R genes they possessed.

Gel electrophoresis pattern of few NRVs for certain markers are shown in Fig 2. The genetic frequencies of the twelve major rice blast resistance genes varied from 0 to 100%. The scoring data indicated that frequency of the positive allele of *R* gene ranges from four genes (9) to twelve genes (1) in the 80 NRVs (Fig 3). The Swarna-Sub1 possesses maximum number of the positive allele of twelve resistance genes. Nine NRVs (11.25%) showed positive bands for only four *R* genes, two (3.12%) were positive for five genes, twelve (15%) for six genes, eighteen (22.50%) for seven genes, thirteen (16.25%) for eight genes, eighteen (22.50%) for nine *R* genes, five (6.25%) for ten genes, two (3.12%) for eleven genes and one (1.25%) for twelve *R* genes (S1 Table). The presence of *Pib* gene was determined by visualization of amplicons of 388 bp fragments using SNP marker Pb28 along with the positive control IRBLB-b. This gene was found to be present in all the NRVs showed gene frequency of 100%. The presence of *Pi9* blast resistance genes was determined by visualization of amplicons of 2000 bp (195R-1), 128 bp (Pi9-pro) and 291 bp (Pi9-i) fragments corresponding to the positive control IRBL9-w. The gene frequency of *Pi9* was found to be 0% with Pi9-pro, 3.75% with Pi9-i and 18.75% with 195R-1 markers. Estimation of PCR results for the *Pi2* genes was determined by visualization of amplicons of 2344 bp (Pi2GM), 399 bp (2LRR) and 450 bp (Pi2-i) fragments respectively corresponding to the positive control IRBLZ5-CA. The gene frequency of *Pi2* was found to be 0% with 2LRR marker, 3.75% with Pi2-i marker and 100% with Pi2-GM marker. The SNP primer, z56592, and zt56591 were used for the estimation of *Piz* and *Piz-t* genes by visualization of amplicons of 292 bp and 257 bp, with monogenic lines IRBLZ-Fu and IRBLZT-t. Results showed the presence of *Piz* gene in 73 varieties with a frequency of 91.25% while *Piz-t* was positive in 45 varieties with a genetic frequency of 56.25%. Forty-three varieties showed positive bands for both *Piz* and *Piz-t* genes. Out of the multi-gene family positioned on chromosome 11, presence of *Pik*, *Pik-p*, *Pikm* and *Pik-h* genes were determined by using the SNP marker K39512, K3957, Indel marker k2167 and candidate gene marker respectively. *Pik* gene was detected in 78 varieties, *Pik*-*p* in twenty-nine varieties, *Pikm* in thirty eight varieties, while *Pik-h* was detected in fifty-six varieties corresponding to the positive control IRBLZK-Ka, IRBLZKP-k60, IRBLKMTS and IRBLKH-k3 with a gene frequency of 97.50%, 36.25%, 47.5% and 70%, respectively. Only two varieties, Ketekijoha and Lunishree were negative for *Pik* gene. Thirteen varieties were positive for above four genes.

For the presence of *Pita/Pita-2* allele, the present result showed that twenty-six (32.50%) varieties produced positive bands of 861 bp with Pita3 primer equivalent to the monogenic line IRBLTA-KI and positive bands of 1,042 bp with YL155/87 primer pairs which were tightly linked to the resistant *Pita*/*Pita*-2 allele. The presence of *Pi1* was scored by visualization of amplicon of 170 bp positive fragments using SSR primer, RM1233. The results showed that only twenty varieties produced positive bands for *Pi1* gene along with positive check C101LAC with a gene frequency of 25%. To know the presence of *Pi5* gene, the 40N23r primer was used, which produces an amplicon of 700 bp in the resistant genotype corresponding to the positive check IRBLz5-M. *Pi5* gene was scored in twenty-six varieties with a genetic frequency of 32.50% (S1 Table). All the genes were amplified using CO39 as a negative control.

The allele numbers per locus varied from 1 to 2 with an average of 1.76, while allele frequency varied from 0.52 to 1.0. The polymorphism information content (PIC) was used to measure the information content of a genetic marker. The PIC value for seventeen varied from 0 (Pb28 and Pi2-GM) to 0.37 (zt56591 and K2167) and the gene diversity ranged from 0 (Pb28 and Pi2-GM) to 0.49 (zt56591 and K2167). The PIC value of markers Pb28 for *Pib* gene and Pi2-GM for *Pi2* gene showed zero due to monomorphic allelism in all the 80 NRVs. On the other hand, the marker zt56591 and K2167 for *Piz-t* and *Pikm* genes showed highest PIC value of 0.37 and therefore is more informative for genetic diversity analysis (S1 Table).

### Cluster analysis

**Fig 4:**
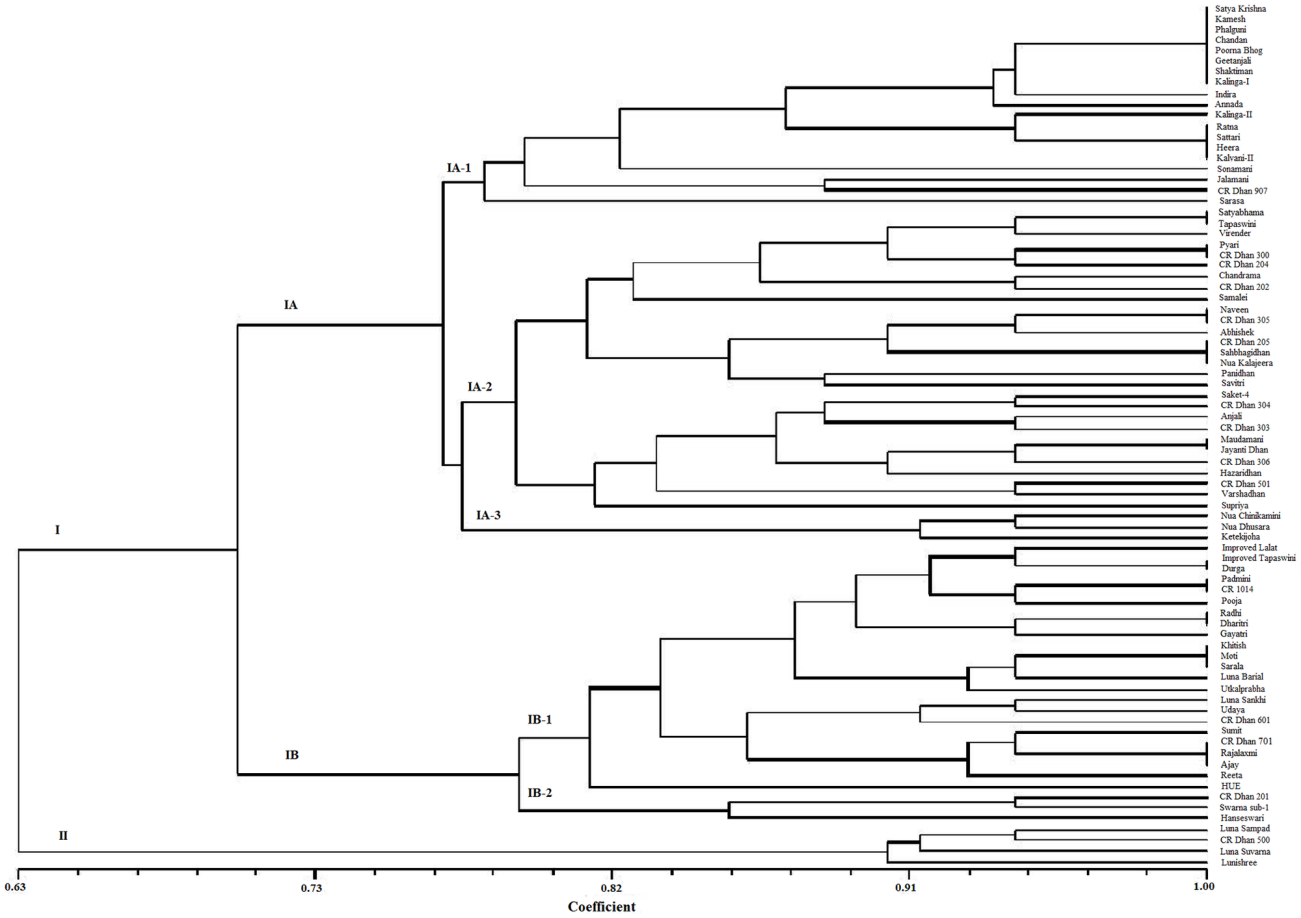
Clustered analysis of 80 NRVs based on R genes.

The eighty NRVs were categorized into two clusters (I and II) at 63% level of genetic similarity coefficient (Fig 4). Major cluster I which included 76 NRVs, was divided into two sub-clusters IA and IB. Further, sub-cluster IA consisting of 50 NRVs was divided into three sub-cluster IA-1, IA-2 and IA-3. Sub-cluster IA-1 consists of 19 NRVs, of which, four (21.05%) are highly resistant. Sub-cluster IA-2 contained 28 NRVs, in which twelve (42.85%) are highly resistant. Cluster IA-3 included only 3 NRVs, with no resistant genotype. Similarly, sub-cluster IB consisting of 26 NRVs was divided into two sub-clusters, IB-1 and IB-2. Sub-clusters IB-1 consisted of 23 NRVs, with only two resistant genotypes (8.33%). On the other hand, sub-clusters IB-2 consisted of only three NRVs but none were found to be resistant. Therefore, IB-1 is composed of susceptible varieites. Cluster II comprised of the remaining 4 NRVs, with only one resistant variety. Interestingly, most of the resistant varieties were grouped in major cluster I. The NRVs of similar ecologies did not belong to the same cluster. In contrast, the genetically similar genotypes of each cluster were characterized by NRVs of different ecologies. Interestingly, Sub-clusters IB-2 included only 3 NRVs, all were susceptible, but with a maximum number of resistance genes (S2 Table).

### Genetic association of blast resistant genes

Genetic association analysis of the blast resistant genes were conducted using the generalized linear model (GLM) to find out any significance association of the blast disease. Out of the seventeen markers used in the present study, only five markers (195R-1, Pi9-i, Pita3, YL155/YL87 and 40N23r) corresponded to three genes (*Pi9*, *Pita/Pita2* and *Pi5)* and found significantly associated with the blast disease (Table 3). The phenotypic variance of these five markers ranged from 3.5% to 7.7%. Out of these five markers, YL155/YL87 of *Pita/Pita-2* gene showed the largest phenotypic variance (7.7%) followed by 40N23r of *Pi5* gene with phenotypic variance of 6.8%. Another markers of the *Pita/Pita-2* gene, Pita3 also showed strong significant association with the phenotypic variance of 5.6%. The gene *Pi9* showed a phenotypic variance of 5.2% and 3.5% with Pi9-i and 195R-1 respectively, while the rest of the markers for nine resistance genes didn’t show significant association at *P* value < 0.1.

**Table 3:** Association of rice blast resistant genes with blast disease in 80 NRVs.

S. No.	Blast *R* Genes	Gene-based/linked markers	*P*-value	*R*^2^_Marker
1	*Pib*	Pb28	0	0
2	*Piz*	Z56592	0.52089	0.0053
3	*Piz-t*	Zt56591	0.14226	0.0274
4	*Pik*	K39512	0.2083	0.02022
5	*Pik-p*	K3957	0.15064	0.0263
6	*Pik-h*	Pikh	0.71191	0.00176
7	***Pi9***	**195R-1**	0.09653	0.03501*
		**Pi9-Pro**	0	0
		**Pi9-i**	0.04017	0.05288*
8	*Pi2*	Pi2GM	0	0
		Pi2-i	0.88756	0.0002579
		2LRR	0	0
9	***Pita/Pita-2***	**Pita3**	0.03386	0.05643**
10	***Pita/Pita-2***	**YL155/YL87**	0.01262	0.07713**
11	*Pi1*	RM1233	0.32725	0.01231
12	***Pi5***	**40N23R**	0.019198	0.06833**
13	***Pikm***	**K2167**	0.11187	0.03209

* and ** denotes significance level at *P* value < 0.1 and 0.05 respectively

### Population structure analysis

**Fig 5:**
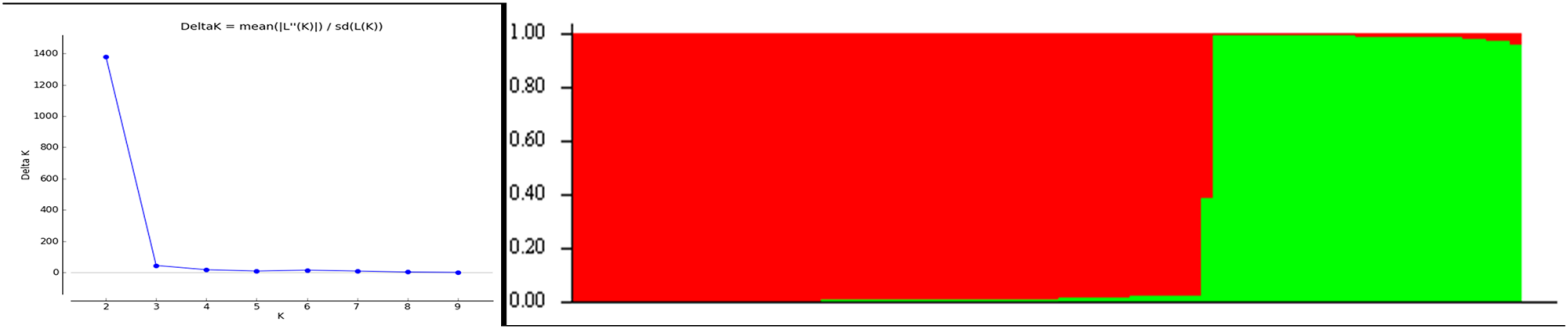
Population structure of 80 NRVs based on 17 markers (K = 2) and graph of estimated membership fraction for K = 2. The maximum of adhoc measure ΔK determined by structure harvester was found to be K = 2, which indicated that the entire population can be grouped into two subgroups. Different color within group indicates the proportion of shared ancestry with other group which has the same color with the admixture.

All the 80 NRVs evaluated for estimation of population structure for blast disease based on 17 markers corresponded to 12 blast resistance genes using Structure software. The peak plateau of adhoc measure ΔK was found to be K = 2 (Fig 5), which indicated that the entire 80 NRVs were distributed into two subgroups (SG1 and SG2). Based on ancestry threshold of >90%, all the 80 NRVs except one, were classified into two subgroups (S3 Table). The NRV, Sonamani has inferred ancestry value of 39.0% and 61.0% for Q1 and Q2 and hence classified as admixture (AD). The SG1 was smaller group consisting 26 NRVs (32.5%) of which 10 and 16 were found to be resistant and susceptible, respectively. Among the resistant, only two varieties, Sumit and Reeta were found to be highly resistant. On the other hand, the SG2 included 53 NRVs (66.2%) of which 28 and 25 NRVs were found to be resistant and susceptible, respectively. There were more numbers of resistant genotypes (28) in the SG2 cluster compared to SG1. The most resistant variety, Sarasa was included in the SG2 cluster. Out of the 29 resistant genotypes in the SG2 cluster, 17 genotypes were found to be highly resistant. If we consider the classification of 80 genotypes into only two groups such as higly resistant (HR; score 0–3) and susceptible (MR & SS; score 4–9), then there are only two HR genotypes out of 26 varieties in SG1 as compared to seventeen HR genotypes out of 53 varieties in SG2. Therefore, the subgroup SG1 can be considered as dominated by susceptible genotypes whereas the subgroup SG2 consisted mostly of highly resistant genotypes. Thus in general, the results indicated that the structure analysis was able to differentiate HR and susceptible into two different separate groups, SG2 and SG1.

**Fig 6:**
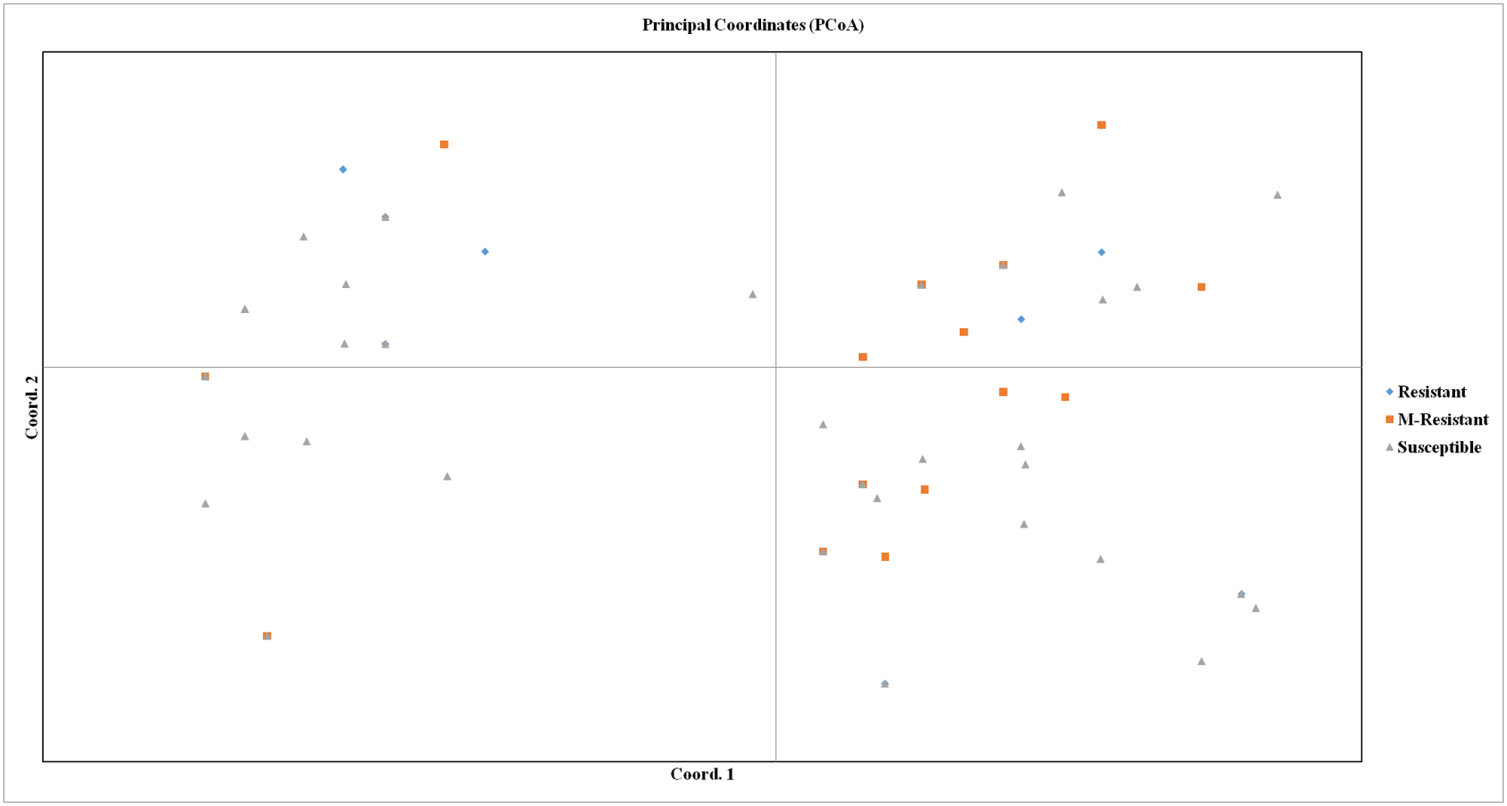
Two dimensional PCoA display of 80 NRVs based on 17 markers. Coord 1 and Coord 2 represent first and second coordinates, respectively. The two PCA axes accounted for 27.55% and 18.40% of the genetic variation among populations.

To establish the genetic relationships of the 80 NRVs based on the 17 markers related to 12 blast *R* genes, the PCoA was further constructed. A scatter plot generated from the PCoA analysis showed that the first two components accounted for 24.34% and 17.04% of the genetic variation amounting a total of 41.38% of genetic variation (Fig 6). These scatter plots also showed a clear separation of 80 NRVs into two subgroups.

### Estimation of population genetics through AMOVA analysis

In order to conduct the AMOVA analysis, the 80 NRVs were grouped according to their blast disease score as HR; highly resistant (score 0–3), MR; moderate resistant (score 4–5) and HS; highly susceptible (score 6–9). Based on this criteria, the 80 NRVs were distributed into three populations; HR (19), MR (21) and HS (40). It was found that more variance (96%) was observed within population,whereas between population it was less (4%) (Table 4). The pair-wise fixation indices (F_ST_) among the populations were given in the Table 5. The highest pair wise F_ST_ was observed between the highly resistant and susceptible whereas, the lowest was found to be between the highly resistant and moderately resistant populations. Based on the estimated value of fixation indices, it is indicated that, there is weak population structure and they aren’t genetically isolated from each other.

**Table 4:** Results of analysis of molecular variance (AMOVA).

Source	df	SS	MS	Est. Var.	Percentage of variation
**Among Populations**	2	18.075	9.037	0.098	4%
**Within populations**	77	320.349	4.160	2.080	96%
**Total**	79	338.425	-	2.178	100%

df: degree of freedom; SS: sum of squares; MS: mean squares; Est. Var. estimated variance (p > 0.001)

**Table 5:** Pair-wise F_ST_ estimates among the three populations of NRVs.

Populations	Highly resistant	Moderate resistant	Susceptible
**Highly resistant**	0.000		
**Moderate resistant**	0.040	0.000	
**Susceptible**	0.073	0.052	0.000

Fst values below diagonal

## Discussion

In the phenotype-based screening, it is often difficult to detect the presence of individual resistance genes,as it is influenced by the developmental stage and environmental conditions. DNA markers tightly linked to resistance genes offer an efficient and quickway to choose for many types of blast resistance genes without performing phenotype-based screening [30]. In the present study, eighty NRVs spreaded over eight different ecologies were phenotyped and genotyped for twelve major blast resistance genes which could provide valuable information for using in gene pyramiding and gene deployment in different rice growing regions of India for blast disease control and breakdown of *R* genes and can be directly used as a variety or donor for MAS.

Some of the varieties like Satya Krishna, Chandan, and Jalamani contain four resistance genes but still showed resistance. Similarly, Betiga and Chinnaponni landraces showed moderate resistance reaction but did not contain any of the tested genes [31]. Although these varieties had few *R* genes, they may possess other *R*-genes which provide resistance or the resistance might be provided by major gene combines with other major quantitative trait loci or interaction between different minor *R* genes [31].

Swarna-Sub1 was found to contain twelve while Luna Barial and CR Dhan 201 found to harbor eleven resistance genes but still showed susceptible reaction. Similarly, though Udaya, Hanseswari, Moti, Dharitri, and CR Dhan 501 were found positive for more than eight genes, still showed susceptible reaction to the prevalent races at Cuttack. Similar results were also reported previously that in some cases the presence of several genes did not ensure resistance [32]. This variation in phenotypic result may be due to the presence of a type of an allele in those lines [31]. The characterization of gene function will allow the development of allele-specific markers that would be more efficient than linked DNA markers [33].

Marker-assisted selection (MAS) is a classical tool in breeding for improved resistance to rice blast. For MAS, the selection is made based on DNA markers closely linked to a blast *R* gene that confers resistance to a particular race of the pathogen [34]. In the present study, genetic frequencies of the 12 major rice blast resistance genes, *Pib*, *Piz*, *Piz*-*t*, *Pik*, *Pik*-*p*, *Pikm*, *Pik-h*, *Pita/Pita-2*, *Pi2*, *Pi9*, *Pi1*, and *Pi5* varied from 0 to 100%. Similarly, the gene frequency of the nine major rice blast resistance genes varied from 6 to 97% in the in North East and Eastern germplasm and the genetic frequencies of the 10 major rice blast resistance genes ranged from 19.79 to 54.69% [32,35].

The *Pib* gene appeared to be ubiquitous were detected in the all the eighty NRVs. Similar results were also reported in North East and Eastern germplasm and Manipur rice accessions [32,36]. The presence of *Pi*9 gene was detected in 15 NRVs (18.75%). The *Pi*9 gene was also reported in two North East and Eastern Indian rice germplasm and six rice germplasm among 47 rice accessions [32,37] but it was not detected in Manipur accessions [36]. One of the reasons for the rare occurrence of *Pi*9 gene in the *indica* rice is its introgression from the wild species O. *minuta* [37]. Surprisingly, all the NRVs were found positive for *Pi2* gene using gene specific primer Pi-2GM. In another study, 60 landraces harbored *Pi2* gene with a genetic frequency of 72.28% [31]. The result of *Piz and Piz-t* genes showed its presence in seventy three and forty-five varieties. However, they confer only partial resistance to the varieties tested. Similarly, they did not contribute to complete resistance in any of the germplasms evaluated [32]. Of the multi-gene family positioned on chromosome 11, *Pik-h* gene,which was originally isolated from *indica* variety Tetep [38]. It was present in fifty-six NR varieties (70%). In another study, *Pik-h* gene were found in 18 and 52 accessions [32,35]. Similarly, *Pik* and *Pik*-*p* and *Pikm* genes has also appeared in seventy eight, twenty nine and thirty eight NRVs, respectively. Imam et al. [32] also reported the *Pik* gene as the most frequently detected gene in their study. The two blast resistance genes, *Pita* and *Pita*-2, are tightly linked to each other and located near the centromere on chromosome 12 [39]. Identification and validation of *Pi-ta* genes in the Indian rice germplasm reveal that this rice germplasm is diverse and could be used as a potential donor for developing blast resistant lines [40]. The present result showed that only twenty-six (32.50%) varieties were positive for Pita3 and dominant marker YL155/YL87. Similar study was carried out by several researchers in recent past showing the genetic frequency of 19.29%, 6.25% and 27% in indica genotypes [35,32,40]. The results of *Pi1* and *Pi5* gene showed positive bands in twenty and twenty-six NRVs of 170 and 700 bp amplicon corresponding to the positive control C101LAC and IRBLz5-M. Similarly, *Pi1* gene was detected in 39 landraces with a frequency of 46.98% [31]. In another study, 60 landraces (72.28%) and 4 (18.2%) landraces from Manipur showed the presence of *Pi5* gene [31,36].

The present study categorized eighty NRVs into two group at 63% level of genetic similarity. Our results showed that the genetically similar genotypes of each cluster were differentiated by NRVs of different ecologies. Similarly, the population structure for eighty NRVs based on seventeen markers classified them into two subgroups (SG1 and SG2). The smaller group SG1 consists of 26 (32.50%) NRVs, of which, most of them are susceptible genotypes and only two resistant variety whereas, the major group SG2 included 53 NRVs (66.25%) of which 17 are resistant genotypes. Accordingly, through structure analysis eighty NRVs were differentiated into two groups containing susceptible and resistant genotypes. The corresponding NRVs in SG1 and SG2 were found concurrent with the sub-cluster IB and IA respectively. On the other hand, remaining four NRVs of major cluster II were found in subgroup G2. Likewise, through PCoA analysis, the scatter plots represent a partition of 80 NRVs into two subgroups. Association mapping is an important strategy used for identifying genes controlling important traits which has been successfully used for diagnosis of human diseases [41]. Association study of blast resistance in indica rice and finger millet blast resistant genes showed its importance in identification of markers linked to the loci or QTls conferring blast resistance [42,43]. In this study, only five markers such as 195R-1, Pi9-i, Pita3, YL155/YL87 and 40N23r corresponded to three genes viz. *Pi9*, *Pita/Pita2* and *Pi5* which were established to be considerably associated with the blast disease. The consistent results showed by the individual markers for selected blast resistance genes makes them a suitable marker for genotyping of rice blast resistant genes in the rice germplasm. Moreover, the association between the number of resistance gene(s) and the disease reaction were not completely understood in our study which could be explained by addition of more number of markers or these varieties needs to be tested for new resistance genes/alleles or QTls.

Durable resistance refers to resistance that remains effective during its prolonged and widespread use in environments favorable to the pathogen or disease spread [44]. Here, at least five years resistance after its release is considered as durable. In our study, 19 were HR, of which 15 NRVs were documented as either resistant or moderate resistant to blast disease during the release to public (S4 Table). While for remaining 4 NRVs, exact documents are not available about their release with blast resistance; only it was mentioned that they showed tolerance to abiotic and biotic stresses. Out of nineteen varieties, 11 varieties released between 1980–2010 were found as durable NRVs (Table 6). The durability of these 11 NRVs for blast disease varied from 5 to 35 years.

**Table 6:** Durability of resistant NRVs against leaf blast.

S.N.	NRVs	Released		No. of *R* genes	Blast disease reaction (score) at present	Durability (years)
		In (Year)	With			
1	Satya Krishna	2008	R- NBl,	4	HR (1)	7
2	Satyabhama	2012	MR-BL	7	HR (3)	3
3	Chandan	2008	MR-BL	4	HR (2)	7
4	Chandrama	2006	R-Blast	6	HR (1)	9
5	Abhishek	2006	R-BL	8	HR (1)	9
6	CR Dhan 205	2014	Leaf Blast	9	HR (1)	1
7	CR Dhan 202	2014	MR- LB	7	HR (2)	1
8	CR Dhan 204	2014	MR to leaf blast	7	HR (1)	1
9	Sahbhagidhan	2009	Res. to leaf blast	9	HR (1)	6
10	Sarasa	1985	-	6	HR (0)	30
11	CR Dhan 300	2013	MR-LB, NBL	6	HR (2)	2
12	Jalamani	2012	MR-BL, NBL	4	HR (2)	3
13	Sumit	2012	R-LB	9	HR (2)	3
14	Jayanti Dhan	2012	MR- BL, NBL	9	HR (2)	3
15	Panidhan	1988	Tol. to biotic stress	9	HR (1)	27
16	Savitri	1982	Tol. to biotic stress	9	HR (1)	33
17	Reeta	2010	R- LB; MR- NBL	9	HR (1)	5
18	Samalei	1980	R-blast	7	HR (1)	35
19	Lunishree	1992	Tol. to biotic & abiotic stress	7	HR (1)	23

R- resistant; MR- moderate resistant; BL- blast; NBL-neck blast; Tol-Tolerant; HR-Highly resistant.

Among the 19 resistant NRVs, only five varieties (Sarasa, Panidhan, Samalei, Savitri and Lunishree), released during 1990s showed their effectiveness and durability to the different races of *M*. *oryzae* over a long period. Samalei showed maximum durability for thirty five years. The *R* gene in these resistant NRVs varied from four to nine (S2 Fig). Similarly, NRVs released between 2006–2014 still showed resistance to *M*. *oryzae*. Interestingly, most of the varieties found resistant were released as resistant to moderarely resistant and some showed field tolerance to major diseases and pests.

## Conclusion

Phenotyping of NRVs against leaf blast and molecular screening and genetic diversity for major blast resistance genes will help in rational use of these varieties. The present study provided an overview of the genetic diversity of the eighty rice varieties for leaf blast resistance. Besides, the accurate evaluation of blast resistance genes in rice varieties, and the marker loci obtained are highly informative and efficient in the selection of parental lines and development of new breeding populations. The information obtained from the phenotypic reaction and genetic variability of the varieties will be very much useful for proper selection of varieties in different blast prone areas and could also be utilized in gene deployment and gene pyramiding on the basis of prevalence of *M*. *oryzae* races. Additionally, some of the varieties possessed other blast resistance genes, could be used in mapping of genes and in the application of marker assisted selection. Being adapted to particular ecologies, and having coevolved with the local population of the blast fungus, these resistant varieties will be possibly exploited which have more advantages over other foreign resistance donors currently being used in the breeding programs. Moreover, these varieties have been bred with good agronomic characteristics along with multiple disease and insect resistance and abiotic stress tolerance.

## Supporting information

S1 Fig Loci of different markers used in this study. (TIF)

S2 Fig Number of resistance genes present in the 80 NRVs. (TIF)

S1 Table SNPs, STS and SSR markers associated with (number) blast resistance genes, gene frequency, gene diversity and PIC value in NRRI released varieties and their reaction to leaf blast in uniform blast nursery. (XLSX)

S2 Table Clustering pattern of NRVs based on gene based PCR amplification. (XLSX)

S3 Table Population structure group of 80 NRVs based on inferred ancestry values. (XLSX)

S4 Table Detailed information of rice varieties released by National Rice Research Instititue, Cuttack. (XLSX)
