# Correction: Use of molecular markers in identification and characterization of resistance to rice blast in India

**DOI:** 10.1371/journal.pone.0179467

**Published:** 2017-06-07

**Authors:** Manoj Kumar Yadav, Aravindan S., Umakanta Ngangkham, H. N. Subudhi, Manas Kumar Bag, Totan Adak, Sushmita Munda, Sanghamitra Samantaray, Mayabini Jena

The fourth author’s name is spelled incorrectly. The correct name is: H. N. Subudhi. The correct citation is: Yadav MK, S. A, Ngangkham U, Subudhi HN, Bag MK, Adak T, et al. (2017) Use of molecular markers in identification and characterization of resistance to rice blast in India. PLoS ONE 12(4): e0176236. https://doi.org/10.1371/journal.pone.0176236
