# Supplementary figures and images for: Use of molecular markers in identification and characterization of resistance to rice blast in India
Source: PLoS One. 2017 Apr 26;12(4):e0176236. doi: 10.1371/journal.pone.0176236 (PMC5405977; doi:10.1371/journal.pone.0176236)

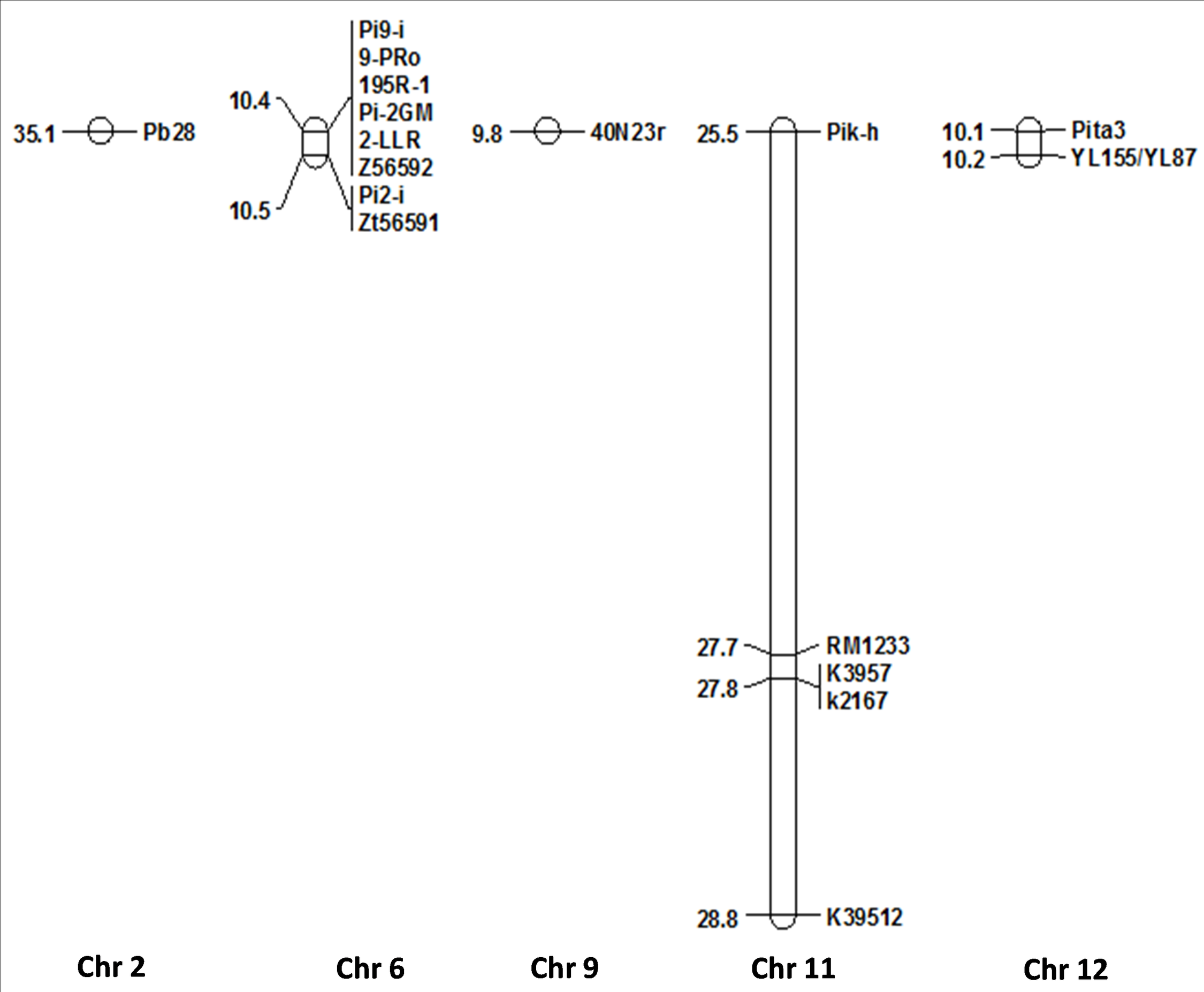

Supplement: S1 Fig — (TIF) [file pone.0176236.s001.tif]

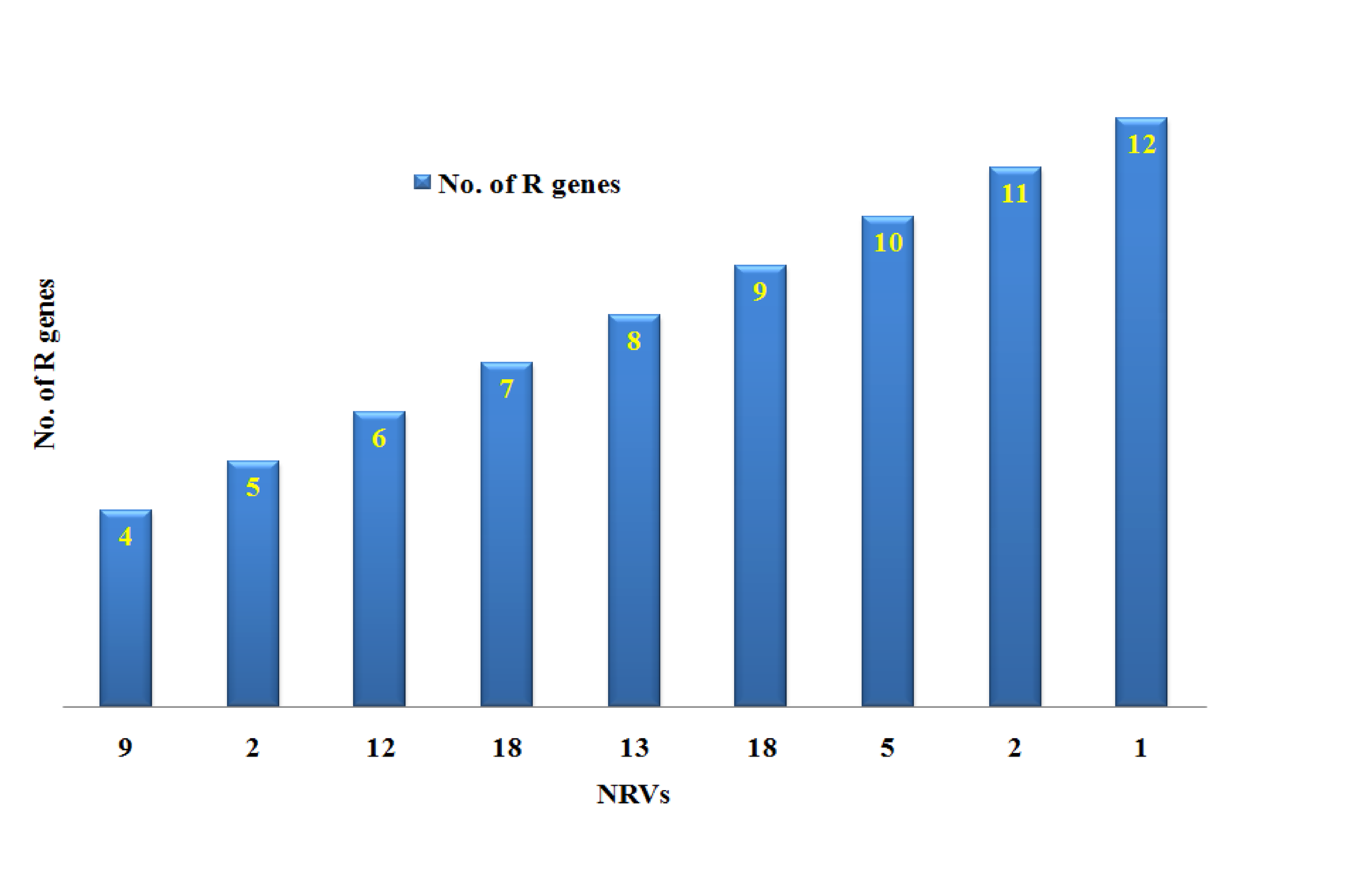

Supplement: S2 Fig — (TIF) [file pone.0176236.s002.tif]
